# Editorial: Optical imaging and laser technologies in neuro-oncology

**DOI:** 10.3389/fonc.2022.1103711

**Published:** 2023-01-10

**Authors:** Konstantin Yashin, Tatiana Novikova, Vladislav Shcheslavskiy

**Affiliations:** ^1^ Department of Oncology and Neurosurgery, University Clinic, Privolzhsky Research Medical University, Nizhny Novgorod, Russia; ^2^ LPICM, CNRS, École Polytechnique, Institut Polytechnique de Paris, Palaiseau, France; ^3^ Department of Biomedical Engineering, Florida International University, Miami, FL, United States; ^4^ Institute of Experimental Oncology and Biomedical Technologies, Privolzhsky Research Medical University, Nizhny Novgorod, Russia

**Keywords:** optical imaging, fluorescence imaging, confocal microscopy, optical coherence tomography, Raman spectroscopy, polarization imaging, optical biopsy

Use of light in neuro-oncology can be twofold, for either diagnosis or therapeutics. The optical bioimaging technologies, such as confocal microscopy, multiphoton tomography, optical coherence tomography, Raman spectroscopy, fluorescence and polarization imaging are the most trending methods currently to guide surgical procedures and intraoperative “optical” biopsy. On the other side, using lasers as the surgical tools in the interstitial thermal therapy and photodynamic therapy can provide targeted destruction of brain tumor cells. Moreover, the combination of diagnostic and therapeutic applications of light opens the avenue for the new developments in biomedical engineering.

In this Frontiers issue, “*Optical imaging and Laser technologies in Neuro-Oncology*”, we are privileged to present a collection of 8 open-access publications that describe the recent advances in research and practice of using optical technologies in neuro-oncology. These articles were selected through an open peer-review process that unites experts in the field.

The first group of articles advances our knowledge in the field of fluorescence imaging that is widely used in neurosurgical oncology. The presented clinical studies address the usefulness of 5-ALA guidance during awake speech mapping for augmenting the extent of resection for infiltrative high-grade gliomas and identifying foci of anaplasia in non-enhancing gliomas (Goryaynov et al.) and usefulness of fluorescein guidance for pilocytic astrocytomas for improving the extent of resection (Falco et al.).

The second group of papers relays information on novel brain tumor optical imaging techniques including papers on Raman imaging (Romanishkin et al.), confocal laser endomicroscopy with fluoresceint sodium (Xu et al.), optical coherence tomography (Strenge et al.), and polarization imaging technique (Liu et al.). We must note, though, that the confocal laser endomicroscopy with fluorescein sodium described technologies are not FDA-approved and are still under research at the various stages of development.

All these techniques aim at better intraoperative visualization of tumors to improve the accuracy and extent of resection. However, their individual limitations require the development of the multimodal approach based on the combination of several techniques providing comprehensive information derived from the different sources regarding biological, metabolic, anatomical, and functional properties of brain tissue. Multimodal imaging concept improves the extent of resection while preserving functional non-tumor tissue, thus, overcoming the limitations of each specific method.

For the optical imaging techniques quantitative, as opposed to qualitative assessment, requires more scrutiny. The implementation of deep learning methods for high accuracy diagnosis of leptomeningeal metastasis, based on the analysis of cells from cerebrospinal fluid has demonstrated the utility of deep learning in the classification of different tissue characteristics with optical imaging (Yu et al.).

Optical imaging has also a great potential in supporting diagnostic and therapeutic approaches in neuro-oncology based on tumor models. The development of a patient-derived glioblastoma model that steadily co-expresses luciferase and a far-red fluorescent protein in combination with the optical imaging techniques - macroscopic fluorescence lifetime imaging (macro-FLIM) and cross-polarization optical coherence tomography (CP OCT) provides an excellent approach for studying tumor biology or developing novel therapeutic strategies (Yuzhakova et al.).

This constellation of basic, translational, and clinical studies provides a comprehensive review of optical imaging technologies used in neuro-oncology, soundly demonstrates their utility and supports the necessity of the further progress in the development of optically based technologies for neuro-oncology. The extensive work performed by the authors and other colleagues promotes and advances the new optical techniques as a toolkit for everyone working at the intersection of neurosurgery, oncology, and biomedical optical imaging.

## Author contributions

All authors listed have made a substantial, direct and intellectual contribution to the work, and approved it for publication, all authors agree to be accountable for the content of the work.

